# PPAR*γ*2^Pro12Ala^ Polymorphism and Human Health

**DOI:** 10.1155/2009/849538

**Published:** 2009-04-16

**Authors:** Weimin He

**Affiliations:** Alkek Institute of Biosciences and Technology, Texas A&M University System Health Science Center, 2121 W. Holcombe Blvd., Houston, TX 77030, USA

## Abstract

The nuclear hormone receptor peroxisome proliferator activated receptor gamma (PPAR*γ*) is an important transcription factor regulating adipocyte differentiation, lipid and glucose homeostasis, and insulin sensitivity. Numerous genetic mutations of PPAR*γ* have been identified and these mutations positively or negatively regulate insulin sensitivity. Among these, a relatively common polymorphism of PPAR*γ*, Pro12Ala of PPAR*γ*2, the isoform expressed only in adipose tissue has been shown to be associated with lower body mass index, enhanced insulin sensitivity, and resistance to the risk of type 2 diabetes in human subjects carrying this mutation. Subsequent studies in different ethnic populations, however, have revealed conflicting results, suggesting a complex interaction between the PPAR*γ*2 Pro12Ala polymorphism and environmental factors such as the ratio of dietary unsaturated fatty acids to saturated fatty acids and/or between the PPAR*γ*2 Pro12Ala polymorphism and genetic factors such as polymorphic mutations in other genes. In addition, this polymorphic mutation in PPAR*γ*2 is associated with other aspects of human diseases, including cancers, polycystic ovary syndrome, Alzheimer disease and aging. This review will highlight findings from recent studies.

## 1. Introduction

Peroxisome proliferator activator receptor gamma (PPAR*γ*) is a member of the nuclear hormone receptor superfamily that transcriptionally regulates genes controlling a variety of biological functions including cell growth, differentiation, and metabolism in response to lipophilic hormones, dietary fatty acids, and their metabolites [1]. Unlike some steroid hormone receptors such as the estrogen receptor, that are bound by heat shock proteins and sequestered in the cytoplasm, PPAR*γ* is constitutively localized in the nucleus [2], heterodimerizes with the retinoid X receptor (RXR) [3], and binds to corepressors [4]. Ligand binding results in a conformational change in the receptor, triggering dissociation of corepressor complex and recruitment of coactivator proteins, leading to activation of gene expression [4]. 

**Figure 1:**
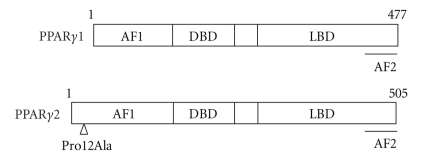
Domain structure of human PPAR*γ*. AF1, activation function 1; DBD, DNA binding domain; LBD, ligand binding domain; AF2, activation function 2.

Human PPAR*γ* gene is located in chromosome 3 and spans a genomic segment of >150 kb. It consists of 9 exons (A1, A2, B, and 1–6), from which the two distinct isoforms of PPAR*γ* mRNA and protein, PPAR*γ*1 and PPAR*γ*2, are derived through the use of separate promoters and 5*'* exons. PPAR*γ*1 mRNA specie is comprised of exons A1, A2, and 1–6, and is translated from P1 promoter while PPAR*γ*2 mRNA is a combination of exons B and 1–6 and is translated from P2 promoter. The two proteins differ by the presence of extra 28 amino acids at the NH_2_-terminus of PPAR*γ*2 [5, 6]. PPAR*γ* is abundantly expressed in adipose tissue, colon and macrophages while its expression is much lower in skeletal muscle, heart and other tissues [7, 8]. PPAR*γ*1 is ubiquitously expressed whereas PPAR*γ*2 expression is restricted to adipose tissue [9] (Figure 1). 

**Figure 2:**
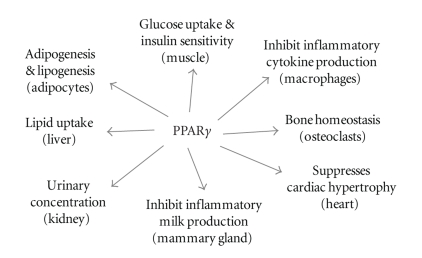
Pleiotropic functions of PPAR*γ* in different organs/tissues.

PPAR*γ* plays many functional roles in different organs and tissues (Figure 2). In vivo and in vitro studies demonstrate its critical role in regulating adipocyte differentiation and promoting lipid accumulation in adipose tissue [10–13]. It is also important for maintaining the viability and normal function of differentiated adipocytes [14–16]. In macrophages, PPAR*γ* may enhance foam cell formation and atherogenesis upon increased uptake of oxidized low-density lipoprotein (oxLDL) [17, 18] or increases liver X receptor (LXR)-ATP-binding cassette A1 (ABCA1)-dependent cholesterol efflux upon pharmacological activation by its agonist TZDs [19, 20]. PPAR*γ* in macrophages has also been shown to be involved in suppression of inflammatory cytokine production [21, 22] and improvement of insulin sensitivity [23, 24]. PPAR*γ* in Skeletal muscle critically regulates normal glucose metabolism in muscle and lipid homeostasis in fat and the liver [25, 26] while PPAR*γ* in the liver is implicated in controlling systemic glucose and lipid metabolism [27, 28]. PPAR*γ* also plays roles in regulating bone homeostasis [29], heart hypertrophy [30, 31], high fat diet-induced hypertension [32], and urine concentration in the kidney (Cao et al., unpublished data). 

**Figure 3:**
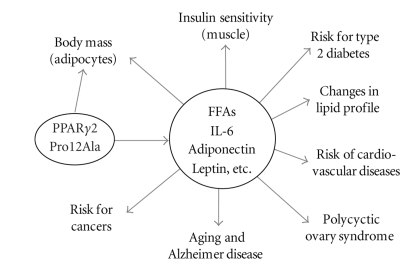
Effects of PPAR*γ*2^Pro12Ala^ polymorphism on various aspects of human health. FFAs, free fatty acids, IL-6, interleukin 6.

PPAR*γ* is also intimately implicated in regulation of glucose and lipid homeostasis and insulin sensitivity [33–35]. Not surprisingly, PPAR*γ* has been identified as the target for thiazolidinediones (TZDs) [36], a class of synthetic compounds that improve insulin sensitivity in a variety of insulin resistant animal models and diabetic patients [33–35]. This role of PPAR*γ* in affecting insulin action is consistent with many human genetic studies with various single amino acid mutations, including Pro12Ala, Pro115 Gln, Cys114Arg, Cyc131Tyr, Cyc162Trp, Val290Met, Pro388Leu, Arg425Cyc, His477His, and Pro467Leu that are scattered in activation function domain 1 (AF1), DNA binding domain (DBD), or ligand binding domain (LBD) of the receptor [37–45]. These mutations result in either gain-of-function or loss-of-function of the receptor; human subjects bearing these mutations show decreased or increased lipid accumulation in adipose tissue, enhanced insulin sensitivity or insulin resistance, dyslipidemia, diabetes, and hypertension [46–50]. Among these, Pro12Ala mutation in PPAR*γ*2 (PPAR*γ*2^ Pro12Ala ^) is the most common. This mutation was first identified by Schuldiner's group in 1997 [37], with different ethnic populations showing various allelic frequencies. Caucasians have the highest frequency (12%), followed by Mexican Americans (10%), West Samoans (8%), African Americans (3%) while Chinese have the lowest (1%) [37]. In the last 10 years, extensive studies have been undertaken to assess the effects of this polymorphism on many aspects of human physiology (Figure 3). This review will summarize the effect of this mutation on human health revealed in these studies.

## 2. Effect of PPAR*γ*2^Pro12Ala^ on Adiposity

Soon after the identification of Pro12Ala mutation, an independent study demonstrates that Ala12 variant is associated with decreased transactivation function of PPAR*γ*2 and lower body mass index (BMI) [51]. This finding is consistent with reduced adipogenic function of the mutant receptor in 3T3-L1 preadipocytes [52]. However, further studies in various ethnic populations demonstrate that effect of this mutation on body mass is more complex. An association of Ala12 variant with decreased adiposity is confirmed in diabetic, nondiabetic, or healthy subjects [53–58]. In studies involving an African American population and a white American population, this mutation is associated with lower BMI in African Americans and increased BMI in white Americans [59, 60], indicating that same genetic mutation results in different responses in different ethnic groups. PPAR*γ*2^ Pro12Ala^ mutation has been also shown to enhance weight loss brought about by exercise in offspring of type 2 diabetic subject [61] or prevent body weight regain after weight loss [62–64]. However, numerous studies suggests an association of Ala12 variant with increased risk of obesity, including studies in ethnic populations of Mexican Americans [65], male Spanish adults [66] or Spanish children and adolescents [67], French [68], male white Italians [69], French Canadians [70], male Brazilians of European descent [71], native Javanese [72], Uygurs, Kazaks, Hans (Chinese) [73], and Greek young girls [74]. This association can also be found in nondiabetic and nonobese or obese Americans [75], obese Finnish women [76], overweight Korean female subjects [but not in lean female subjects] [77] and in Turkish women with gestational diabetes [78]. In addition, women with the Ala12 allele have also been shown to gain more weight than women with Pro12 allele [79]. Despite these, studies in Germans [80], French [81], Hispanics (Colorado, US) [82], Japanese [83], Koreans [84, 85] and Polish [86] do not show an association between Pro12Ala polymorphism and body fat mass. Meta-analysis of 57 studies on nondiabetic individuals show that Caucasians with the X (Pro or Ala)/12Ala genotype is associated with significantly increased BMI, although no difference can be found in the global population [87]. These results indicate that a mild change in PPAR*γ*2 transcription activity has a significant impact on lipid accumulation in adipose tissue. 

It is unclear how a single genetic mutation results in conflict results in different ethnic populations. Given the proadipogenic role of PPAR*γ*, it can be expected that moderate reduction of PPAR*γ*2 transactivation function results in lower BMI in PPAR*γ*2^ Pro12Ala^ carriers. Heterogeneous effects of this polymorphic mutation on adiposity in association studies clearly show that PPAR*γ* regulation of human adipose tissue physiology is a complex process. Several studies suggest roles of genetic or environmental contexts, such as the character of the diet, in shaping the patterns of associations of Pro12Ala polymorphism with body fat composition in different human populations. In at least two studies, ratio of dietary polyunsaturated fatty acid to saturated fatty acid (P : S ratio) have been shown to significantly affects body mass in Ala12 allele carriers. Thus, intake of a diet with higher P : S ratio results in lower BMI while a food with lower P : S ratio is inversely associated with BMI in human subjects carrying Ala12 allele [88]. Similarly, intake of monounsaturated fatty acid also shows such an effect in Ala12 allele carriers [89]. In another study, total fat and saturated fat intake is positively correlated with body mass change in Pro12 homozygotes while Ala12 allele carriers are protected [70]. In addition, changes in genetic context, such as coexistence of other polymorphisms, may have a significant impact on the effect of Pro12Ala polymorphism on body weight composition, resulting opposite findings mentioned above (flip-flop phenomenon) [90]. For example, either Pro12Ala or G174C (promoter region) of interleukin 6 (IL-6) shows an effect on reducing body fat mass or preventing body weight regain after weight loss and the presence of both variants has an additive effect [54, 62]. On the other hand, subjects bearing both Pro12Ala and Trp64Arg of *β*_3_-adrenergic receptor (*β*_3_-AR^ Trp64Arg ^) have increased risk to obesity when compared to those carrying only a single mutation in a case-control study [67] or in a study in dizygotic twins [91], while subjects with the Ala12 allele become more obese only when they also carry the Trp64Arg variant in a Mexican American population [92]. These data suggest complex interactions between genes that both affect lipid metabolism. Yet, there is no study thus far to show that the effect of Pro12Ala polymorphism is negated by mutations in other genes.

## 3. PPAR*γ*2^Pro12Ala^ Regulating Insulin Sensitivity

PPAR*γ*2^ Pro12Ala^ has also been found to increase insulin sensitivity in middle-aged and elderly Finns [51] and this finding is confirmed by subsequent studies in other populations, assessed by plasma levels of insulin and homeostasis model assessment of insulin resistance (HOMA-IR) [54, 55, 59, 66, 79, 93–95]. In healthy carriers of the Ala12 allele, second-phase insulin secretion in response to free fatty acid infusion or insulin secretion in response to arginine is significantly decreased compared to subjects with Pro12 genotype [96]. Although increased glucose uptake in skeletal muscle is observed only in lean but not in obese subjects in Finns carrying Ala12 allele [97], enhanced insulin sensitivity is observed in obese children [98, 99] as well as in obese adults [100, 101]. Even in diabetic patients, Ala12 allele is associate with lower fasting insulin and increased insulin sensitivity [102], more significant hypoglycemic effect of exercise [103], and increased response to TZD treatment [104]. A population-based study in twins also shows a significant impact of the Ala12 allele on maintaining glucose tolerance and insulin sensitivity [105]. Meta-analysis of such studies confirmed a significantly lower levels of fasting insulin in subjects with the homozygous Ala12Ala genotype compared to the Pro12Pro genotype and significantly greater fasting glucose levels and insulin resistance in obese subjects in the Pro12Pro group [87]. These findings point to a beneficial effect of Ala12 variant on systemic insulin sensitivity. 

The effect of PPAR*γ*2^ Pro12Ala^ polymorphism on insulin sensitivity can be influenced by dietary fatty acids and/or physical activity. Intake of monounsaturated fatty acids is inversely associated with insulin resistance in a Spanish population with Ala12 allele, especially in those with significant obesity [106]. Both dietary P : S ratio and physical activity have been shown to inversely associated with fasting insulin concentration [107]. The effect of dietary P : S ratio on fasting insulin is significant only in physically active, but not in physical inactive subjects carrying Ala12 allele [108]. Ala12 allele also interacts with other genes to influence insulin sensitivity. PPAR*α* Leu162Val allele has been found to be associated with impaired glucose tolerance and this deleterious effect of PPAR*α* mutation is neutralized by the Ala12 variant [109]. Similarly, the Gly > Arg mutation (Gly97Arg) of the insulin receptor substrate 1 (IRS1) is associated with a 15% increased risk of type 2 diabetes, although the difference is not significant [110]. Against this genetic background, insulin sensitivity is almost twice greater in carriers of the 12Ala allele than in subjects with Pro12 allele while no such effect of Ala12 allele can be seen on the Gly97 background [111]. Such a protective effect of Ala12 allele on insulin sensitivity can also be observed in human subjects carrying both the Ala12 allele and the Lys121Gln polymorphism of plasma cell 1 (PC-1) glycoprotein [112]. Subjects bearing PC-1^ Lys121Gln^ variant show higher levels of fasting glucose and decreased insulin sensitivity on Pro12 background, whereas this effect of PC-1^ Lys121Gln^ variant is lost on Ala12 background [113]. These results further support the notion that PPAR*γ*2^ Pro12Ala^ polymorphism interacts with other genetic mutations to affect systemic insulin sensitivity and glucose homeostasis.

## 4. Association of PPAR*γ*2^Pro12Ala^ with the Risk of Type II Diabetes

A large-scale family-based study shows an association between Pro12Ala mutation and reduced risk of type 2 diabetes (T2D) [110]. A similar result is obtained in twins carrying Ala12 allele [105]. However, further studies clearly show heterogeneous effects of this polymorphism on predicting susceptibility to the risk of diabetes in various populations. Resistance to the risk of diabetes has been found in Ala12 allele carriers compared to Pro12 allele carriers in ethnic populations as diverse as Japanese [114–116], Korean [117], Iranians [118], Scotts [119], Danish [120], Finns [121], French [122], Spanish [106], and American Caucasians [123, 124]. On the other hand, Ala12 allele has also shown to be functional leading to a predisposition to T2D in populations of Germans [125, 126], Finns [127], Italians [128], Dutch [129], US Caucasians [130], French Caucasians [81], British/Irish Caucasians [131], Asian Indians (Sikh) [132], Parkateje Indians [133], and Arabians [134]. Again, no such effect of Ala12 on the risk of type 2 diabetes can be observed in such diverse populations of Italians [135], Tunisians [136], Qatarians [137], Polish [138], and non-Hispanic and Hispanic white women [139]. In spite of such heterogeneity, however, meta-analysis of these studies indicates that Ala12 carriers have an average of 19% reduced risk of T2D compared to Pro12 carriers. BMI seems to be a major factor accountable for the heterogeneous effect of Pro12Ala polymorphism on the risk for T2D since the risk reduction is greater when BMI is lower. Risk reduction is higher in Asians carrying Ala12 allele (35%) than in Northern Americans and Europeans with the Ala12 genotype (18% and 15%, resp.) compared to their own Pro12 allele controls. When adjusted for the BMI of controls, difference between Asians and Europeans is no longer significant. Even among Europeans, Northern Europeans carrying Ala12 allele show significantly reduced risk for T2D (26%) while the risk reduction in Central and Southern Europeans with Ala12 allele is barely significantly (10%) or is not significant at all (0%) [140]. These data suggest a generally beneficial role of Ala12 allele in preventing the pathogenesis of T2D in several populations with lower body fat mass. 

While the heterogeneity between Asians and other populations is statistically explained by BMI, this is not the case for the heterogeneity observed in Europeans, indicating that other factors, including different genetic and/or environmental background might cause the heterogeneous Pro12Ala-related T2D risk in Europeans. Indeed, the protective role of Ala12 allele against T2D is considerably affected by dietary lipid levels. In a study in human subjects from Ethiopia, Benin, Ecuador, Italy, and world populations, protection against T2D can be observed mainly in populations where energy from lipids exceeds 30% of total energy intake [141]. However, lipid composition in the diet is a significant determination factor since chronic intake of *trans* fatty acids and saturated fatty acids predispose to increased risk of T2D and impaired fasting glucose in Ala12 carriers than Pro12 carriers [142]. In addition, intrauterine condition may also determine the risk of T2D in later life. A study in Dutch population suggests that subjects bearing Ala12 allele are associated with a higher prevalence of impaired glucose tolerance and T2D when they are prenatally exposed to famine during midgestation [129]. On the other hand, Finns carrying Ala12 allele who have smaller body weight at birth seem to be protected against insulin resistance and T2D [143]. Again, Pro12Ala polymorphism interacts with other genetic mutations to affect the risk of developing diabetes. Subjects with the Ala12Ala allele and Gly972Gly variant of IRS-1 have significantly higher plasma adiponectin levels compared to those with the Pro12Pro and Gly972Gly genotype [144]. In Mexican Americans, subjects with the Ala12 allele become more obese only when they also carry the Trp64Arg of Beta-3 adrenergic receptor (*β*-3AR^ Trp64Arg ^) polymorphism [92]. In a study in dizygotic twin pairs, those with both *β*-3AR^ Trp64Arg^ and PPAR*γ*2^ Pro12Ala^ polymorphisms show greater BMI, waist to hip ratio, percent of body fat, and blood glucose [91]. Such interaction between the two polymorphisms also increases the risk of obesity in children and adolescents [67]. In a family-based study in Chinese and Japanese, subjects with both Ala12 allele and the adiponectin T allele are more insulin sensitive than subjects bearing other combinations of genotypes [145]. Recently, an interaction between Ala12 variant and a single nucleotide polymorphism of PPAR*δ* (rs6902123) has been found to contribute to conversion from impaired glucose tolerance to T2D [121]. These studies again emphasize the importance of taking into account of other gene mutations when determining an effect of Pro12Ala polymorphism on the risk of T2D.

## 5. Effect on Other Components of Metabolic Syndrome

The Ala12 allele has been shown to be associated with reduced prevalence of essential hypertension in Chinese nonagenarians and centenarian [146]. Ala12 allele carriers also show lower blood pressure than subjects carrying Pro12 allele [120, 147] and the Ala12 allele is associated with lower diastolic blood pressure in male, but not in female subjects with T2D [148]. Furthermore, hypertensive subjects with lower birth weight or shorter length at birth and Pro12Pro variant have raised blood systolic blood pressure [149]. However, others have suggested either a potential contribution of Ala12 variant to hypertension [115] or an association of Ala12 allele with higher diastolic blood pressure in obese patients with T2D [150] while couple of studies fails to show an association between the PPAR*γ*2 variant and hypertension [151, 152].

Triglyceride (TAG) and cholesterol metabolism may be regulated by Pro12Ala mutation. Ala12 allele is inversely associated with blood TAG concentrations in one report [54] while it has also been found to be associated with a trend of an increase in TAG and hyperlipidemia in another [152]. This variant has also been shown to be associated with lower levels of serum total and nonhigh-density lipoprotein (non-HDL)-cholesterol in a general population [153], lower low-density lipoprotein (LDL)-cholesterol in T2D patients [154], or higher levels of serum HDL-cholesterol in family-based or population-based studies [155, 156]. however, several studies also show an association of Ala12 allele with higher concentration of low-density lipoprotein (LDL)-cholesterol [68, 157] and lower HDL-cholesterol [70]. Interestingly, Pro12Ala mutation interacts with body size at birth to modulate cholesterol metabolism since an association between increased concentration of serum total, LDL- and non-HDL-cholesterol and Ala12 allele can be found only in those who had birth weights below 3 kilograms [158]. In addition, cholesterol metabolism is also affected by genotype-alcohol interaction since Ala12 allele carriers consuming alcohol have higher serum total and HDL cholesterol while the nondrinkers carrying Ala12 allele show lower serum total and HDL cholesterol compared with Pro12 homozygotes [155]. 

Due to its role in regulating lipid metabolism, Pro12Ala polymorphism may influence risk of cardiovascular complications such as atherosclerosis and coronary artery diseases. Ala12 allele does not seem to affect the risk of acute myocardial infarction, coronary artery disease, and ischemic stroke in healthy subjects [159, 160]. In a population with an increased risk of T2D and cardiovascular disease, however, improvement in flow-mediated vasodilation and reduction of serum C-reactive protein (CRP), a risk factor for cardiovascular disease, are prominent only in Ala12 allele carriers, but not in Pro12 homozygotes [161]. Consistently, Ala12 allele carriers have been found to have lower carotid intima-media thickness [162, 163] and decreased risk of myocardial infarction [164] in T2D patients. Yet again, studies do show that Ala12 allele either is associated with increased risk of myocardial infarction [165, 166], or attenuates the protective effect of polyunsaturated fatty acids on myocardial infarction [167], or confers excess hazard of developing cardiovascular diseases in patients with diabetic nephropathy [168]. 

As a result of affecting lipid homeostasis and risk of diabetes, Pro12Ala mutation can be expected to influence diabetic complications. Notably, Ala12 allele is associated with decreased risk of developing diabetic nephropathy compared to Pro12 allele in a case-control study [169]. Ala12 allele carriers also have significantly reduced urinary albumin excretion than noncarriers and the reduction becomes even more dramatic along with increased duration of diabetes [154, 170]. Ala12 variant has also been shown to be associated with decreased risk of diabetic retinopathy in T2D patients [171]. These data suggest a protective effect of the Ala12 allele in relation to complications associated with T2D.

## 6. Effect on Polycystic Ovary Syndrome

Central obesity, insulin resistance, and hyperinsulinemia are typical feature of polycystic ovary syndrome (PCOS) and significant number of PCOS patients show impaired glucose tolerance and are in increased risk of developing T2D [172]. Studies show that frequency of Ala12 allele is significantly reduced in the PCOS group compared with the control group [173, 174]. Moreover, PCOS subjects carrying Ala12 allele show lower levels of free sex hormones (testosterone, androstenedione, and dehydroepiandrosterone sulfate) and reduced luteinizing hormone/follicle-stimulating hormone ratio compared to PCOS subjects carrying Pro12 allele [174]. Insulin sensitivity, evidenced by fasting insulin and HOMA-IR, is also significantly improved in Ala12 allele carriers than in Pro12 allele carriers [174–177]. Even in first-degree relatives of PCOS subjects, distribution of Ala12 Allele is significantly reduced compared to Pro12 allele [178] and fasting insulin and HOMA-IR are lower in first-degree relatives of PCOS subjects with Ala12 variant compared to first-degree relatives of PCOS subjects with Pro12 allele [178].

## 7. Cellular Mechanism of PPAR*γ*2^Pro12Ala^ Polymorphism

Since PPAR*γ*2 is expressed only in adipose tissue, how moderate reduction of PPAR*γ*2 activity in adipose tissue influences insulin sensitivity, diabetes, and other metabolic parameters have been studied but not fully elucidated. Given the role of adipose tissue free fatty acids and adipokines in regulating insulin sensitivity, the effect of Pro12Ala polymorphism can be anticipated to be mediated by changes in these factors. Indeed, subjects with Ala12 allele show lower lipoprotein lipase (LPL) activity [179], which may result in decreased breakdown of lipoproteins and hence, reduced plasma FFAs, which is deleterious to insulin action in skeletal muscle [180]. Consistent with this, Ala12 allele carriers have lower plasma FFAs, higher adipose tissue and skeletal muscle blood flow, and greater insulin-mediated postprandial hormone-sensitive lipase suppression along with greater insulin sensitivity [181]. Besides, insulin suppression of lipolysis in adipose tissue is also increased in lean subjects or in T2D patients carrying Ala12 allele than in subjects with Pro12Pro allele [182, 183]. However, long-term inhibition of lipolysis will, in theory, result in increased adiposity (body mass) rather than lean phenotype in Ala12 allele carriers. Indeed, one study suggests there is an association between Ala12 allele and increased body mass [182]. Obviously, this may not be the true mechanism or may not be the only mechanism underlying the effect of Pro12Ala. Adipose-derived cytokines leptin and adiponectin levels have been shown to increase insulin action [184, 185]. Indeed, Ala12 allele is associated with higher plasma levels of leptin in Spanish diabetic women [186]. In comparison, two Japanese population studies show that Ala12 allele carriers have significantly lower plasma levels of adiponectin than Pro12 allele carriers [187, 188] and another two case-control studies in either diabetic patients or women with PCOS fail to find significant change in serum adiponectin levels [189, 190]. Adiponectin does not seem to play a role in increasing insulin sensitivity in Ala12 allele carriers. Finally, recent studies suggest that increased oxidative stress in adipose tissue is a contributing factor to insulin resistance in obesity [191] and that insulin sensitization by PPAR*γ* agonists is mediated, at least in part, by suppressing oxidative stress in adipose tissue [192]. In adipose tissue-restricted PPAR*γ* heterozygous mice that show reduction of PPAR*γ* in adipose tissue and similarly increased insulin sensitivity as in human subjects carrying Ala12 allele, antioxidant genes are significantly increased; this may be associated with increased resistance to chemical-induced oxidative stress in these animals [193]. Yet, it has not been investigated whether Pro12Ala polymorphism of PPAR*γ*2 is associated with changes in oxidative stress in adipose tissue thus far.

## 8. PPAR*γ*2^Pro12Ala^ Polymorphism and Risk of Cancers

PPAR*γ* ligands have been shown to inhibit proliferation of many tumor cells in vitro and PPAR*γ* may also be implicated in tumorigenesis in vivo [194]. Although PPAR*γ*2 is exclusively expressed in adipose tissue, genetic variation of PPAR*γ*2 seems to indirectly affect the risk of several forms of tumors. The most studied thus far is the association between Ala12 allele with the risk of colorectal cancer. The Ala12 variant is inversely associated with incident sporadic colorectal adenoma, and the effect of this mutation is especially pronounced in women and those who do not take nonsteroidal anti-inflammatory drugs [195]. In a case-control study, Ala12 allele, together with high lutein intake, low refinery grain intake and a high prudent diet score, is associated with reduced risk of colon cancer [196]. Interestingly, the same study shows an increased rectal cancer risk in Ala12 carriers [196]. In another case-control study, Pro12Pro genotype is associated with increased risk of colorectal cancer while no such association is observed among Ala12 carriers [197]. In comparison, there is no evidence to show a significant association of Ala12 allele with colorectal cancer in an Indian (Asia) population [198]. In 3 studies related to gastric cancer, Ala12 allele has been found to be associated with increased risk of gastric cancer [199–201] and this effect of PPAR*γ* is probably related to gastric mucosa atrophy and *Helipobacteria pylori* infection since the presence of Ala12 allele does not increase the risk of gastric cancer in *H. pylori*-negative subjects [199]. In two studies on prostate cancer, one study finds a 2-fold greater risk of prostate cancer in Ala12 allele carriers with BMI above 27.2 kg/m^2^ compared to those with the Pro12 allele [202] while the other study fails to notice such an association [203]. In addition, a marginally significant increase in the risk of breast cancer is observed in women carrying Ala12 variant [204], but Ala12 allele may decrease the risk of breast cancer associated with alcohol consumption [205]. Finally, Ala12 variant is associated with reduced risk of bladder cancer [206] and renal cell carcinoma [207]. The reason underlying some of the inconsistent findings is unclear, but again may reflect a possibility of gene-gene interaction. In at least one study, Pro12Ala allele interacts with vitamin D receptor (VDR)/bsm/polyA to increase risk of rectal cancer [208].

## 9. Effect on Aging and Alzheimer Disease

The potential role of genetic variability at Pro/Ala loci of PPAR*γ*2 gene on longevity is studied in a group of centenarians and long-lived men show an increased frequency of Pro/Ala genotype [209]. PPAR*γ* may also be associated with Alzheimer disease (AD) since activation of PPAR*γ* decreases the release of amyloid-*β* (A*β*), main component of the amyloid plaques associated with AD [210–212]. In line with these observations, a study shows significant overrepresentation of Ala12 allele in octogenarian AD patients, compared to Pro12 allele [213]. However, this result is in contrast with a reported role of Ala12 variant in protecting pathogenesis of AD in female, but not in male subjects in a case-control study [214], while two studies fail to show an association between the Ala12 variant with the genetic risk of AD [215, 216]. Nevertheless, Ala12 allele carriers show an earlier onset of dementia [215], suggesting that Ala12 allele may modify the age of onset in late-onset AD. Ala12 allele carriers also show increased risk of dementia or cognitive impairment without dementia than noncarriers in diabetic patients [217, 218]. It is unclear how PPAR*γ*2^ Pro12Ala^ polymorphism confers such effects on human lifespan or age-related diseases since a change in PPAR*γ* activity by this mutation is supposed to happen only in adipose tissue. Indeed, preliminary studies suggest that the effect of Ala12 allele on human aging may be attributable to decreased IL-6 levels, although there are also reports that healthy elderly have higher levels of IL-6 [219, 220]. In addition, PPAR*γ*2^ Pro12Ala^ polymorphism may affect pathogenesis of AD by modulating cholesterol metabolism since cholesterol levels influence AD pathology [221, 222]. Studies in larger population are required to further elaborate the role of PPAR*γ*2^ Pro12Ala^ polymorphism on blood cholesterol metabolism and AD.

## 10. Conclusion

Much has been done to evaluate the association between PPAR*γ*2^ Pro12Ala^ polymorphism and body mass, insulin sensitivity, risk of T2D, cancer, and other aspects of human health. However, it is not fully understood how reduction of PPAR*γ* activity in adipose tissue can have such diverse effects on human health. While alteration of fatty acid and cytokine release from adipose tissue may underlie the effect of this mutation on insulin sensitivity and the risk of T2D, it is hard to believe that these factors also account for the effect of Pro12Ala polymorphism on cancer and age-related disease. It is likely that some factors that are overlooked or some unknown factors from adipose tissue may also play a role. Besides, the conflicting results often observed in association studies clearly show the presence of gene-gene interaction. Future association studies should employ a more comprehensive approach, such as linkage disequilibrium or haplotype analyses [223, 224], to examine influence of variants at other genetic loci that may compromise or enhance allelic effect of a genetic polymorphism. PPAR*γ*2^ Pro12Ala^ polymorphism will be a good model to elucidate how alteration of adipose PPAR*γ* activity affects metabolic program and other aspects of human physiology.
